# Testicular microlithiasis and testicular tumor: a review of the literature

**DOI:** 10.1186/s12610-018-0073-3

**Published:** 2018-07-09

**Authors:** Louis Leblanc, François Lagrange, Pierre Lecoanet, Baptiste Marçon, Pascal Eschwege, Jacques Hubert

**Affiliations:** 1 Department of Urology, CHRU Nancy, Nancy, France; 2 CNRS UMR 7039 CRAN, Lorraine University, Nancy, France; 3 IADI-UL-Inserm (U947), Lorraine University, Nancy, France

**Keywords:** Testicular microlithiasis, Testicular tumor, Testicular cancer, Germ cell tumor, Infertility, Ultrasound

## Abstract

**Introduction:**

There are numerous scientific publications on testicular microlithiasis (TML) detected during ultrasound (US) examination. We wished to update the data.

**Methods:**

PubMed was used to identify original articles published between 1998 and May 2017 describing the association between TML and testicular tumor. Studies were only included if TML was diagnosed by US. Studies were then classified into subgroups according to the following criteria: asymptomatic, symptomatic, infertility, cryptorchidism, family or personal history of testicular cancer, and “no given reason for US”. A Z-Test was used to identify differences within these subgroups. In addition, we identified prospective cohorts of TML patients. Numbers, duration of follow-up, and occurrence of the “testicular tumor” event were recorded for each of them.

**Results:**

One hundred and seventy-five articles were identified, 40 of which were included. Our review has not showed a clear evidence that cryptorchidism associated with TML is a risk factor for testicular tumor. However, there seems to be a correlation between infertility associated with TML and a higher tumor risk. There were not enough studies to confirm a relationship between family or personal history associated with TML and the tumor risk. There was also a correlation with a higher tumor risk for symptomatic associated with TML and “no given reason for US” plus TML groups. However, these groups are assumed to contain bias and caution must be taken regarding conclusions. Regarding the prospective cohort studies, 16 testicular tumors appeared in the follow-up of patients with TML, 13 patients had risk factors.

**Conclusion:**

In cases of TML incidental finding by US with the presence of risk factors (personal history of testicular cancer, testicular atrophy, infertility, cryptorchidism) a consultation with a specialist should be considered. In the absence of risk factors, the occurrence of testicular cancer in patients with TML is similar to the risk of the general population.

## Résumé

**Introduction:**

Il existe de nombreux articles sur les microlithiases testiculaires découvertes au cours d’une échographie. Nous voulions mettre à jour les données.

**Méthodes:**

Pubmed a été utilisé pour identifier des articles originaux traitant de l’association entre les microlithiases testiculaires et les tumeurs testiculaires, de 1998 à mai 2017. Les études ont été incluses si les microlithiases étaient diagnostiquées par échographie. Les études ont été classées en sous-groupes selon certains critères (asymptomatiques, symptomatiques, infertilité, cryptorchidie, antécédents familiaux ou personnels de cancer des testicules, et « absence de motif pour la réalisation de l’échographie »). Nous avons utilisé un Z-test pour indiquer les différences au sein de ces groupes. De plus, nous avons identifié des cohortes prospectives de patients atteints de microlithiases testiculaires. Le nombre, la durée du suivi et la survenue de l’événement « tumeur testiculaire » ont été enregistrés pour chacun d’entre eux.

**Résultats:**

Cent soixante-quinze articles ont été identifiés, dont 40 ont été inclus. Dans notre revue, il n’y a pas de lien évident pour que les microlithiases associées à la cryptorchidie soient un facteur de risque de tumeur testiculaire. Il semble y avoir une corrélation entre l’infertilité et les microlithiases avec un risque tumoral plus élevé. Il n’y avait pas assez d’études pour confirmer une relation entre les antécédents personnels ou familiaux associés aux microlithiases et le risque de tumeur. Il y avait aussi une corrélation avec un risque tumoral plus élevé pour les microlithiases dans le groupe symptomatique et le groupe « sans motif de réalisation de l’échographie ». Cependant, ces groupes sont supposés contenir des biais et des précautions doivent être prises en ce qui concerne les conclusions. En ce qui concerne les études de cohorte prospective, 16 tumeurs testiculaires sont apparues dans le suivi des patients atteints de microlithiases. Treize patients avaient des facteurs de risque.

**Conclusion:**

En cas de découverte fortuite de microlithiases testiculaires à l’échographie associées à la présence de facteurs de risque (antécédents personnels de cancer des testicules, atrophie testiculaire, infertilité, cryptorchidie), une consultation avec un spécialiste doit être envisagée. En l’absence de facteurs de risque, la survenue d’un cancer du testicule chez les patients atteints de microlithiases est. similaire au risque pour la population générale.

**Mots-clés:**

microlithiases testiculaires, tumeur testiculaire, cancer testiculaire, tumeur germinale, infertilité, échographie.

## Background

**Fig. 1:**
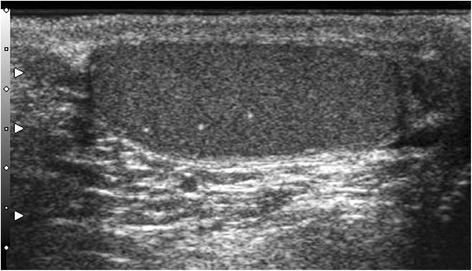
Limited testicular microlithiasis (TML): less than 5 per field of view. With agreement from authors [8]

**Fig. 2:**
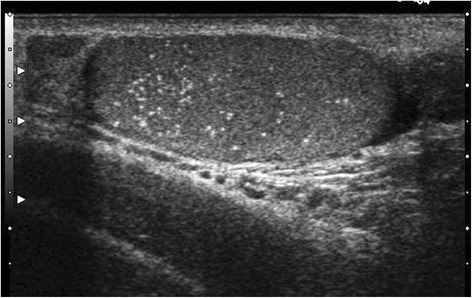
Classic testicular microlithiasis (TML): greater than or equal to 5 per field of view. With agreement from authors [8]

**Fig. 3:**
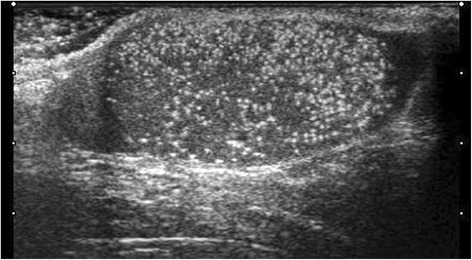
Diffuse testicular microlithiasis (TML). With agreement from authors [8]

Testicular microlithiasis (TML) corresponds to concretions of hydroxyapatite surrounded by fibrosis located in the seminiferous tubes [1]. They are due to the insufficient capacity of Sertoli cells to phagocyte the degenerate cells present in these tubes. They are commonly discovered by ultrasound (US). They are not visible on Magnetic Resonance Imaging (MRI). In 1987, Doherty et al. [2] described their appearance on US, which is characterized by a hyperechoic focus measuring between 1 and 3 mm in the testicular parenchyma without posterior shadow cone [3] with a number greater than or equal to 5 per testis. The discovery is mostly fortuitous because there is no clinical manifestation. Their historical radiological classification is described by Backus et al. [4]. Three grades are distinguished according to the number of TML described by parenchyma (grade 1: 5 to 10, grade 2: 10 to 20 and grade 3 with more than 20 TML). In recent years, US has substantially improved with the advent of higher resolutions enhancing TML detection. In 2015, the European Society of Urogenital Radiology (ESUR) proposed a summary of guidelines and reported another classification with 3 groups, based on the number of TML per field of vision [5]. These three groups were defined as follows, limited TML: less than 5 per field of view (Fig. 1), classic TML: greater than or equal to 5 per field of view (Fig. 2) and finally diffuse TML, labelled “snowstorm” (Fig. 3). There are many observational studies on TML and testicular cancer risk. The objective was to perform a review of the available literature to date.

## Methods

### Search strategy

**Fig. 4:**
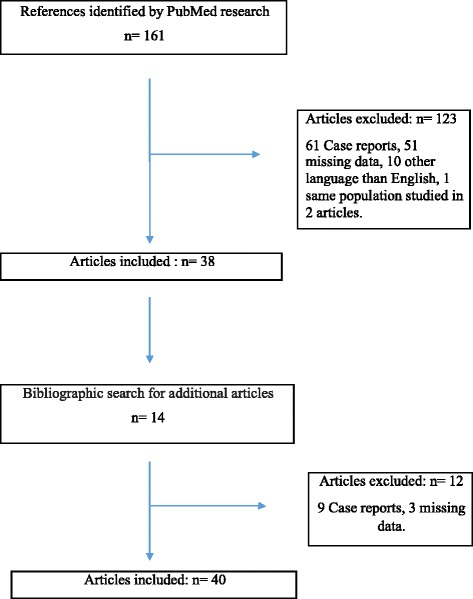
Flowchart and design of the study

The literature review followed the Preferred Reporting Items for Systematic Reviews and Meta-Analyses [PRISMA] guidelines (Fig. 4) [6]. PubMed was used to identify original articles describing the association between TML and testicular tumor, published between 1998 and May 2017. The following keywords were used in the search strategy: testicular microlithiasis, testicular tumor, testicular cancer, testicular neoplasm. Additional studies were included by analyzing the references cited in the review articles. Relevant studies were selected based on the title and abstract.

### Inclusion and exclusion criteria

Studies were included if TML was diagnosed by US. Articles in the English language only were included. Case reports and experimental animal studies were excluded. The following characteristics were collected for each article: year of publication, number of patients included, number of TML carriers, and number of tumor carriers. The studies were classified into subgroups according to the following criteria: asymptomatic, symptomatic, infertility, cryptorchidism, family or personal history of testicular cancer, “no given reason for US”. Finally, prospective cohorts of TML patients were also identified. The number of patients, duration of follow-up and finally the occurrence of the “testicular tumor” event were then recorded for each of them.

### Statistic analyses

We used a Z-Test to indicate differences in these groups. A *P* value of 0.05 or less was considered statistically significant. The analysis was performed with Microsoft Excel 2016 (Microsoft, Seattle, WA, USA).

## Results

One hundred and seventy-five articles were identified. Of these, 119 were excluded by examining the title and abstract (case reports, written in a language other than English). These articles were then read in their entirety. The studies of Yee et al. [7] and Negri et al. [8] included several risk conditions (infertility and cryptorchidism). The same population was found in two articles [9, 10]. A few articles were excluded for missing data.

Finally, a total of 40 articles were selected for our literature review and 135 articles were excluded (Fig. 4).

Two studies concerned asymptomatic cases, 12 concerned symptomatic cases, 11 concerned infertility, 6 concerned cryptorchidism, 2 concerned family or personal history of testicular cancer and 8 referred to “no given reason for US” (Tables 1, 2, 3, 4, 5, 6).

**Table 1:** Asymptomatic cases

Author	Year	*N* = A	Presence of TML		TML Prevalence	No TML	
			Tumor	Total		Tumor	Total
Serter et al. [11] *N* = 2179	2006	2179	0	53	2.4%	0	2926
Peterson et al. [9] *N* = 1504	2001	1504	0	84	5.6%	1	1420
Total		3683	0	137	4%	1	4346

*A* Asymptomatic cases, *N* Number of patients, *TML* Testicular microlithiasis

**Table 2:** Symptomatic population

Author	Year	*N* = S	Presence of TML		TML prevalence	No TML	
			Tumor	Total		Tumor	Total
Pedersen et al. [12] *N* = 1538	2017	1538	8	197	12.8%	25	1358
Richenberg et al. [5] *N* = 2656	2015	2656	0	51	1.9%	0	2605
Volokhina et al. [13] *N* = 2266	2014	2266	1	87	3.8%	8	2179
Deganello et al. [14] *N* = 516	2012	516	1	45	8.7%	0	474
Kosan et al. [15] *N* = 197	2007	197	3	21	10.6%	1	176
Ahmad et al. [16] *N* = 4256	2007	4259	3	32	0.8%	80	4227
Pourbagher et al. [17] *N* = 5263	2005	5263	4	40	0.8%	0	5223
Ringdahl et al. [18] *N* = 160	2004	160	4	12	8%	2	148
Bach et al. [19] *N* = 528	2003	528	12	48	9%	36	480
Middleton et al. [20] *N* = 1079	2002	1079	3	40	3.7%	3	884
Derogee et al. [21] *N* = 1535	2001	1535	30	54	1.8%	31	1472
Skyrme et al. [22] *N* = 2215	2000	2215	5	34	1.4%	24	2181
Total		22,212	74	661	5.3%	210	21,407

*S* Symptomatic population, *N* Number of patients, *TML* Testicular microlithiasis

**Table 3:** Infertility

Author	Year	*N* = I	Presence of TML		TML prevalence	No TML	
			Tumor	Total		Tumor	Total
La Vignera et al. [23] *N* = 1056	2012	320	10	60	18.8%	5	260
Yee et al. [7] *N* = 1429	2011	60	10	10	16.7%	37	50
Negri et al. [8] *N* = 2172	2008	415	12	17	4.1%	2	2029
Sakamoto et al. [24] *N* = 545	2006	545	0	30	5.5%	1	515
Qublan et al. [25] *N* = 384	2006	234	0	23	9.8%	0	211
Mazilli et al. [26] *N* = 303	2005	281	0	13	4.6%	0	268
De Gouveia et al. [27] *N* = 263	2004	263	6 CIS	53	20.1%	1 CIS	210
Von Eckardstein et al. [28] *N* = 1701	2001	1399	22	32	2.3%	61	1367
Thomas et al. [29] *N* = 159	2000	159	0	10	6.3%	0	149
Pierik et al. [30] *N* = 1372	1999	1372	0	12	0.9%	7	1360
Aizenstein et al. [31] *N* = 180	1998	180	0	5	2.8%	0	175
Total		5228	60	265	8.3%	114	6594

*I* Infertilty, *CIS* Carcinoma In Situ, *N* Number of patients, *TML* Testicular microlithiasis

**Table 4:** Cryptorchidism

Author	Year	*N* = C	Presence of TML		TML prevalence	No TML	
			Tumor	Total		Tumor	Total
Cooper et al. [32] *N* = 3370	2014	9	3	9	100%	0	0
Chiang et al. [33] *N* = 31	2012	12	0	12	100%	0	19
Dutra et al. [34] N = 1504	2011	127	0	5	3.9%	0	122
Goede et al. [35] *N* = 501	2010	501	0	14	2.8%	0	487
Konstantinos et al. [36] *N* = 391	2006	36	0	2	5.5%	0	34
Patel et al. [37] *N* = 112	2005	112	0	8	7.1%	0	104
Total		797	3	50	36.5%	0	766

*C* Cryptorchidism, *N* Number of patients, *TML* Testicular microlithiasis

**Table 5:** Family history of testicular tumor

Author	Year	*N* = F	Presence of TML		TML prevalence	No TML	
			Tumor	Total		Tumor	Total
Korde et al. [38] *N* = 81	2008	48	0	23	48%	0	25

*F* Family history, *N* Number of patients, *TML* Testicular microlithiasis

**Table 6:** No given reason for US

Author	Year	*N* = NG	Presence of TML		TML prevalence	No TML	
			Tumor	Total		Tumor	Total
Heller et al. [40] *N* = 6002	2014	6002	53	456	7.6%	84	5546
Chen et al. [41] *N* = 513	2010	513	6	74	14.4%	2	481
Sanli et al. [42] *N* = 4310	2008	4310	17	78	1.8%	58	4232
Miller et al. [43] *N* = 3279	2007	3279	5	67	2%	27	3212
Ou et al. [44] *N* = 1978	2007	1978	9	150	7.6%	17	1828
Lam et al. [45] *N* = 2957	2007	2957	8	137	4.6%	1	137
Otite et al. [46] *N* = 3026	2001	3026	16	54	1.8%	66	2972
Cast et al. [47] *N* = 4892	2000	4892	7	33	0.7%	47	4786
Total		26,957	121	1284	5%	302	23,194

*N* No given reason for US, *N* Number of patients, *TML* Testicular microlithiasis

### Asymptomatic cases

Two studies were identified regarding the asymptomatic population [9, 11], the TML prevalence was 2.4% [11] and 5.6% [9]. Only one testicular tumor was identified in the TML-free population, and no cases were observed in the population with TML. The pooled data revealed no difference in tumor prevalence within the two groups (NS).

### Symptomatic cases

We included 12 studies regarding the symptomatic population [5, 12–22], the TML prevalence was between 0.8% and 12.8%. The criteria for performing US were testicular pain, testicular edema or increased testicular volume. Seventy four cases of testicular tumors were identified in the TML group. Data analysis has shown that testicular tumor prevalence of symptomatic cases with TML was 11.2% and 1% in symptomatic cases TML-free (*p* < 0.0001).

### Infertility

Eleven studies concerned infertility associated with TML [7, 8, 23–31]. In cases of infertility, the TML prevalence varied between 0.9% and 20.1%. Data analysis showed that testicular tumor prevalence was 22.6% in the infertility with TML group versus 1.7% in the infertility TML-free group (p < 0.0001). De Gouveia et al. [27] described a correlation between TML and intratubular germ cell neoplasia by performing a systematic bilateral testicular biopsy in all patients.

### Cryptorchidism

We included 6 studies concerning cryptorchidism [32–37]. Two of these series reported a TML frequency of 100% [32, 33]. Three cases of testicular tumor only were found in the TML population. No testicular tumor was reported in the TML-free population.

### Family or personal history of testicular cancer

One study was found regarding TML associated with family or personal history of testicular tumor, the TML prevalence was 48%. Korde et al. [38] reported that TML was more common in the contralateral testis of men with a personal history of testicular tumor. Coffey et al. [39] was not selected because there was no information on whether patients had TML or not. Bach et al. [19] analyzed the association of TML and contralateral tumor in monorchid patients who underwent contralateral orchidectomy for a testicular tumor. Of the 156 patients examined, 23 had TML (15%). A contralateral testicular tumor was diagnosed in 5 patients with TML (21% versus 2% in the TML-free group).

### No given reason for US

We included 8 studies where there was no given reason for US [40–47]. The prevalence of TML varied between 0.7% to 14.4%. Data analysis showed that the tumor prevalence for “no given reason for US” with TML was 9.4% and 1.3% TML-free (*p* < 0,0001).

### Prospective cohorts of TML

Finally, 16 studies analyzed the occurrence of a “testicular tumor” event in follow-up of patients with TML (Table 7). Out of 1465 patients in total, with a median follow-up of 35.4 months, 16 developed a testicular tumor. Thirteen of the 16 patients had contributing factors. Three patients had a personal history of testicular tumor in the studies by Derogee et al. [21] and Otite et al. [46]. Von Eckardsein et al. [28] reported 2 cases of germ cell tumors involved patients with testicular atrophy. Of the 8 tumor cases reported by Negri et al. [8], 4 patients were infertile and the other 4 had cryptorchidism. Ahmad et al. [16] identified 2 cases of testicular tumor during the follow-up of 29 patients, however no further details were given by the authors. Decastro et al. [10] identified one case of testicular tumor in the follow-up of 63 patients, but no risk factor was reported for this patient.

**Table 7:** Follow-up of patients with TML

Author	Year	Number	Median follow-up	Tumor event
Richenberg et al. [5]	2015	51	33	0
Cooper et al. [32]	2014	83	50	0
Bennet et al. [3]	2011	72	45	0
Negri et al. [8]	2008	835	24	8
DeCastro et al. [10]	2008	63	64	1
Ou et al. [44]	2007	48	29	0
Lam et al. [45]	2007	30	19	0
Kosan et al. [15]	2007	21	19	0
Ahmad et al. [16]	2007	29	40	2
Serter et al. [11]	2006	53	12	0
Sakamoto et al. [24]	2006	32	11	0
Pourbagher et al. [17]	2005	36	34	0
Von Eckardsein et al. [28]	2001	14	48	2
Otite et al. [46]	2001	38	36	2
Derogee et al. [21]	2001	31	62	1
Skyrme et al. [22]	2000	29	41	0

*N* Number of patients, *TML* Testicular microlithiasis, Median follow-up in months; Tumor event: occurrence of the “testicular tumor” event

## Discussion

In recent years, TML have been the source of several epidemiological studies. Older studies reported low TML prevalence: 1.4% [22] and 0.68% [47]. Prevalence is higher in more recent studies: 12.8% [12] and 18.8% [23]. The advent of new generation probes with improved resolution explains this increase. However, there is a higher prevalence in specific populations at risk: patients with cryptorchidism, infertility, family or personal history, testicular tumor. This raises the question of an association between TML and the risk of developing a testicular tumor.

A history of cryptorchidism is a risk factor for testicular cancer [32, 48, 49]. Negri et al. [8] reported a correlation between germ cell tumor and cryptorchidism associated with TML (odds ratio 7,5 *p* = 0,04). In our review, there is no clear evidence showing that TML associated with cryptorchidism is a risk factor for testicular tumor. As only a few studies have shown this association, further research should be carried out to confirm it.

Infertility is a risk factor for testicular cancer [50, 51]. Some studies have assumed a correlation between testicular cancer and infertility associated with TML [38, 52, 53]. Our study seems to confirm a correlation between infertility with TML and a higher tumor risk.

Family or personal history is a risk factor for testicular cancer [54, 55]. In our review, only one study was identified, however no correlation was found between this factor associated with TML and a higher tumor risk. More studies are required to better assess any potential correlation.

In 2016, the literature review by Pedersen et al. [56] showed similar results. TML are not an independent risk factor for testicular cancer. However, when associated with infertility, the risk of testicular tumor increases. Other risk factors identified are McCune-Albright Syndrome and Down Syndrome. Family history of testicular cancer is a risk condition for the presence of TML but not for the risk of testicular cancer.

There are confounding factors regarding the symptomatic group. Some inclusion criteria such as testicular pain, testicular edema or increased testicular volume may reflect the presence of a germ cell tumor and consequently influence the results. These confounding factors are also found in studies in which US is performed without any given indication. Patients included in these cases may have risk factors for testicular tumor.

In a 2015 meta-analysis, Wang et al. [57] concluded that TML have a significant association with testicular cancer. All patients with TML should therefore benefit from close US monitoring. The studies with the most significant forest plot results, Middleton et al. [20], Derogee et al. [21] and Cooper et al. [32], included infertile patients in their samples. The inclusion of studies without distinction of the study population is a confounding factor potentially invalidating the conclusion.

Prospective cohort studies have shown that the occurrence of the testicular tumor event in patients with TML occurred more frequently in patients with testicular cancer risk factors (personal history, infertility, atrophy and cryptorchidism). Patel et al. [58] confirmed the same results in a large retrospective study with a follow-up of 14 years. Among the 442 patients studied, only 2 patients developed a testicular tumor, and both had an independent risk factor of testicular cancer. Furthermore, Pedersen et al. [56] showed that patients often forget to attend their US follow-up. A long term prospective study is difficult to organize.

In 2010, in another meta-analysis, Tan et al. [59] investigated the potential association between TML and intratubular germ cell neoplasia (ITGCN). The study reports a high risk of concomitant discovery of ITGCN and TML when a biopsy is performed on a contralateral testicle of a patient with a history of testicular cancer. ITGCN is where dysplastic cells proliferate inside the seminiferous tubules without crossing the basal membrane. In 2015, Richenberg et al. [5] showed that clustering of TML could cause an unstable area inside the testicle where ITGCN can grow. In patients with a history of orchiectomy for testicular tumors, when TML are present in the contralateral testis, ITGCN is present in 20% of cases. Fifty percent of ITGCN evolve into malignancy within 5 years [60]. A testicular biopsy is then recommended. When an ITGCN is found, therapeutic options can be either external radiotherapy or straight follow up with delayed treatment when a testicular tumor appears. Given the lack of benefit to overall survival, morbidity treatment must be considered, including hypogonadism.

The studies included had different objectives, which may have resulted in selection bias and therefore modify the relationship between TML and testicular cancer. This is the main limitation of the present paper.

We have not studied the histological types of tumor, which may constitute a second bias. Other longitudinal clinical studies should be carried out to determine the association between TML and testicular tumors.

## Conclusion

In cases of TML incidental finding by US with the presence of risk factors (personal history of testicular cancer, testicular atrophy, infertility, cryptorchidism) a consultation with a specialist should be considered. In the absence of risk factors, the occurrence of testicular cancer in patients with TML is similar to the risk of the general population.

## Abbreviations

CIS: Carcinoma In Situ
ITGCN: Intratubular Germ Cell Neoplasia
MRI: Magnetic Resonance Imaging
TML: Testicular microlithiasis
US: Ultrasound

## References

[1] Renshaw AA (1998). Testicular calcifications: incidence, histology and proposed pathological criteria for testicular microlithiasis. J Urol.

[2] Doherty FJ, Mullins TL, Sant GR, Drinkwater MA, Ucci AA (1987). Testicular microlithiasis. A unique sonographic appearance. J Ultrasound Med Off J Am Inst Ultrasound Med.

[3] Bennett HF, Middleton WD, Bullock AD, Teefey SA (2001). Testicular microlithiasis: US follow-up. Radiology.

[4] Backus ML, Mack LA, Middleton WD, King BF, Winter TC, True LD (1994). Testicular microlithiasis: imaging appearances and pathologic correlation. Radiology.

[5] Richenberg J, Belfield J, Ramchandani P, Rocher L, Freeman S, Tsili AC (2015). Testicular microlithiasis imaging and follow-up: guidelines of the ESUR scrotal imaging subcommittee. Eur Radiol.

[6] Moher D, Liberati A, Tetzlaff J, Altman DG, PRISMA Group (2009). Preferred reporting items for systematic reviews and meta-analyses: the PRISMA statement. Open Med Peer-Rev Indep Open-Access J.

[7] Yee WS, Kim YS, Kim SJ, Choi JB, Kim SI, Ahn HS (2011). Testicular microlithiasis: prevalence and clinical significance in a population referred for scrotal ultrasonography. Korean J Urol.

[8] Negri L, Benaglia R, Fiamengo B, Pizzocaro A, Albani E, Levi Setti PE (2008). Cancer risk in male factor-infertility. Placenta.

[9] Peterson AC, Bauman JM, Light DE, McMann LP, Costabile RA (2001). The prevalence of testicular microlithiasis in an asymptomatic population of men 18 to 35 years old. J Urol.

[10] DeCastro BJ, Peterson AC, Costabile RA (2008). A 5-year followup study of asymptomatic men with testicular microlithiasis. J Urol.

[11] Serter S, Gümüş B, Unlü M, Tunçyürek O, Tarhan S, Ayyildiz V (2006). Prevalence of testicular microlithiasis in an asymptomatic population. Scand J Urol Nephrol.

[12] Pedersen MR, Møller H, Rafaelsen SR, Jørgensen MMB, Osther PJ, Vedsted P (2017). Characteristics of symptomatic men with testicular microlithiasis - a Danish cross-sectional questionnaire study. Andrology.

[13] Volokhina YV, Oyoyo UE, Miller JH (2014). Ultrasound demonstration of testicular microlithiasis in pediatric patients: is there an association with testicular germ cell tumors?. Pediatr Radiol.

[14] Deganello A, Svasti-Salee D, Allen P, Clarke JL, Sellars MEK, Sidhu PS (2012). Scrotal calcification in a symptomatic paediatric population: prevalence, location, and appearance in a cohort of 516 patients. Clin Radiol.

[15] Kosan M, Gonulalan U, Ugurlu O, Oztekin V, Akdemir O, Adsan O (2007). Testicular microlithiasis in patients with scrotal symptoms and its relationship to testicular tumors. Urology.

[16] Ahmad I, Krishna NS, Clark R, Nairn R, Al-Saffar N (2007). Testicular microlithiasis: prevalence and risk of concurrent and interval development of testicular tumor in a referred population. Int Urol Nephrol.

[17] Pourbagher MA, Kilinc F, Guvel S, Pourbagher A, Egilmez T, Ozkardes H (2005). Follow-up of testicular microlithiasis for subsequent testicular cancer development. Urol Int.

[18] Ringdahl E, Claybrook K, Teague JL, Northrup M (2004). Testicular microlithiasis and its relation to testicular cancer on ultrasound findings of symptomatic men. J Urol.

[19] Bach AM, Hann LE, Shi W, Giess CS, Yoo H-H, Sheinfeld J (2003). Is there an increased incidence of contralateral testicular cancer in patients with intratesticular microlithiasis?. AJR Am J Roentgenol.

[20] Middleton WD, Teefey SA, Santillan CS (2002). Testicular microlithiasis: prospective analysis of prevalence and associated tumor. Radiology.

[21] Derogee M, Bevers RF, Prins HJ, Jonges TG, Elbers FH, Boon TA (2001). Testicular microlithiasis, a premalignant condition: prevalence, histopathologic findings, and relation to testicular tumor. Urology.

[22] Skyrme RJ, Fenn NJ, Jones AR, Bowsher WG (2000). Testicular microlithiasis in a UK population: its incidence, associations and follow-up. BJU Int.

[23] La Vignera S, Condorelli R, Vicari E, D’Agata R, Calogero AE (2012). Testicular microlithiasis: analysis of prevalence and associated testicular cancer in central-eastern Sicilian andrological patients. Andrologia.

[24] Sakamoto H, Shichizyou T, Saito K, Okumura T, Ogawa Y, Yoshida H (2006). Testicular microlithiasis identified ultrasonographically in Japanese adult patients: prevalence and associated conditions. Urology.

[25] Qublan HS, Al-Okoor K, Al-Ghoweri AS, Abu-Qamar A (2007). Sonographic spectrum of scrotal abnormalities in infertile men. J Clin Ultrasound JCU..

[26] Mazzilli F, Delfino M, Imbrogno N, Elia J, Spinosa V, Di Nardo R (2005). Seminal profile of subjects with testicular microlithiasis and testicular calcifications. Fertil Steril.

[27] de Gouveia Brazao CA, Pierik FH, Oosterhuis JW, Dohle GR, Looijenga LHJ, Weber RFA (2004). Bilateral testicular microlithiasis predicts the presence of the precursor of testicular germ cell tumors in subfertile men. J Urol.

[28] von Eckardstein S, Tsakmakidis G, Kamischke A, Rolf C, Nieschlag E (2001). Sonographic testicular microlithiasis as an indicator of premalignant conditions in normal and infertile men. J Androl.

[29] Thomas K, Wood SJ, Thompson AJ, Pilling D, Lewis-Jones DI (2000). The incidence and significance of testicular microlithiasis in a subfertile population. Br J Radiol.

[30] Pierik FH, Dohle GR, van Muiswinkel JM, Vreeburg JT, Weber RF (1999). Is Routine scrotal ultrasound advantageous in infertile men?. J Urol.

[31] Aizenstein RI, DiDomenico D, Wilbur AC, O’Neil HK (1998). Testicular microlithiasis: association with male infertility. J Clin Ultrasound JCU.

[32] Cooper ML, Kaefer M, Fan R, Rink RC, Jennings SG, Karmazyn B (2014). Testicular microlithiasis in children and associated testicular cancer. Radiology.

[33] Chiang LW, Yap T-L, Asiri MM, Phaik Ong CC, Low Y, Jacobsen AS (2012). Implications of incidental finding of testicular microlithiasis in paediatric patients. J Pediatr Urol.

[34] Dutra RA, Perez-Bóscollo AC, Melo EC, Cruvinel JC (2011). Clinical importance and prevalence of testicular microlithiasis in pediatric patients. Acta Cir Bras.

[35] Goede J, Hack WWM, van der Voort-Doedens LM, Pierik FH, Looijenga LHJ, Sijstermans K (2010). Testicular microlithiasis in boys and young men with congenital or acquired undescended (ascending) testis. J Urol.

[36] Konstantinos S, Alevizos A, Anargiros M, Constantinos M, Athanase H, Konstantinos B (2006). Association between testicular microlithiasis, testicular cancer, cryptorchidism and history of ascending testis. Int Braz J Urol Off J Braz Soc Urol.

[37] Patel RP, Kolon TF, Huff DS, Carr MC, Zderic SA, Canning DA (2005). Testicular microlithiasis and antisperm antibodies following testicular biopsy in boys with cryptorchidism. J Urol.

[38] Korde LA, Premkumar A, Mueller C, Rosenberg P, Soho C, Bratslavsky G (2008). Increased prevalence of testicular microlithiasis in men with familial testicular cancer and their relatives. Br J Cancer.

[39] Coffey J, Huddart RA, Elliott F, Sohaib SA, Parker E, Dudakia D (2007). Testicular microlithiasis as a familial risk factor for testicular germ cell tumour. Br J Cancer.

[40] Heller HT, Oliff MC, Doubilet PM, O’Leary MP, Benson CB (2014). Testicular microlithiasis: prevalence and association with primary testicular neoplasm. J Clin Ultrasound JCU.

[41] Chen J-L, Chou Y-H, Tiu C-M, Chiou H-J, Wang H-K, Chiou S-Y (2010). Testicular microlithiasis: analysis of prevalence and associated testicular cancer in Taiwanese men. J Clin Ultrasound JCU..

[42] Sanli O, Kadioglu A, Atar M, Acar O, Nane I, Kadioglu A (2008). Grading of classical testicular microlithiasis has no effect on the prevalence of associated testicular tumors. Urol Int.

[43] Miller FNAC, Rosairo S, Clarke JL, Sriprasad S, Muir GH, Sidhu PS (2007). Testicular calcification and microlithiasis: association with primary intra-testicular malignancy in 3,477 patients. Eur Radiol.

[44] Ou S-M, Lee S-S, Tang S-H, Wu S-T, Wu C-J, Cha T-L (2007). Testicular microlithiasis in Taiwanese men. Arch Androl.

[45] Lam DL, Gerscovich EO, Kuo MC, McGahan JP (2007). Testicular microlithiasis: our experience of 10 years. J Ultrasound Med Off J Am Inst Ultrasound Med..

[46] Otite U, Webb JA, Oliver RT, Badenoch DF, Nargund VH (2001). Testicular microlithiasis: is it a benign condition with malignant potential?. Eur Urol.

[47] Cast JE, Nelson WM, Early AS, Biyani S, Cooksey G, Warnock NG (2000). Testicular microlithiasis: prevalence and tumor risk in a population referred for scrotal sonography. AJR Am J Roentgenol.

[48] Giwercman A, Grindsted J, Hansen B, Jensen OM, Skakkebaek NE (1987). Testicular cancer risk in boys with maldescended testis: a cohort study. J Urol.

[49] Husmann DA (2005). Cryptorchidism and its relationship to testicular neoplasia and microlithiasis. Urology.

[50] Doria-Rose VP, Biggs ML, Weiss NS (2005). Subfertility and the risk of testicular germ cell tumors (United States). Cancer Causes Control CCC.

[51] Møller H, Skakkebaek NE (1999). Risk of testicular cancer in subfertile men: case-control study. BMJ.

[52] Costabile RA (2007). How worrisome is testicular microlithiasis?. Curr Opin Urol.

[53] van Casteren NJ, Looijenga LHJ, Dohle GR (2009). Testicular microlithiasis and carcinoma in situ overview and proposed clinical guideline. Int J Androl.

[54] Hemminki K, Li X (2004). Familial risk in testicular cancer as a clue to a heritable and environmental aetiology. Br J Cancer.

[55] Heimdal K, Olsson H, Tretli S, Fosså SD, Børresen AL, Bishop DT (1997). A segregation analysis of testicular cancer based on Norwegian and Swedish families. Br J Cancer.

[56] Pedersen MR, Rafaelsen SR, Møller H, Vedsted P, Osther PJ (2016). Testicular microlithiasis and testicular cancer: review of the literature. Int Urol Nephrol.

[57] Wang P-Y, Shen M-Y (2009). Testicular microlithiasis: ultrasonic diagnosis and correlation with male infertility. Zhonghua Nan Ke Xue Natl J Androl.

[58] Patel KV, Navaratne S, Barlett E, Clarke JL, Muir GH, Sellars ME (2016). Testicular microlithiasis: is sonographic surveillance necessary? Single Centre 14 year experience in 442 patients with testicular microlithiasis. Ultraschall Med.

[59] Tan IB, Ang KK, Ching BC, Mohan C, Toh CK, Tan MH (2010). Testicular microlithiasis predicts concurrent testicular germ cell tumors and intratubular germ cell neoplasia of unclassified type in adults: a meta-analysis and systematic review. Cancer.

[60] von der Maase H, Rørth M, Walbom-Jørgensen S, Sørensen BL, Christophersen IS, Hald T (1986). Carcinoma in situ of contralateral testis in patients with testicular germ cell cancer: study of 27 cases in 500 patients. Br Med J Clin Res Ed.

